# Heat Tolerance in *Magallana hongkongensis*: Integrative Analysis of DNA Damage, Antioxidant Defense, and Stress Gene Regulation

**DOI:** 10.3390/antiox14091075

**Published:** 2025-09-02

**Authors:** Tuo Yao, Xiaodi Wang, Jie Lu, Shengli Fu, Changhong Cheng, Lingtong Ye

**Affiliations:** 1 Key Laboratory of South China Sea Fishery Resources Exploitation & Utilization, Ministry of Agriculture and Rural Affairs, South China Sea Fisheries Research Institute, Chinese Academy of Fishery Sciences, Guangzhou 510300, China; yaotuo@scsfri.ac.cn (T.Y.); xiaodiwang1205@yahoo.com (X.W.); lujie@scsfri.ac.cn (J.L.); fushengli@scsfri.ac.cn (S.F.); 2 Sanya Tropical Fisheries Research Institute, Sanya 572018, China; 3 Tropical Aquaculture Research and Development Center, South China Sea Fisheries Research Institute, Chinese Academy of Fishery Sciences, Sanya 572018, China; 4 Shenzhen Base of South China Sea Fisheries Research Institute, Chinese Academy of Fishery Sciences, Shenzhen 518121, China; 5 College of Fisheries and Life Science, Dalian Ocean University, Dalian 116023, China

**Keywords:** *Magallana hongkongensis*, heat stress, antioxidant defense, heat shock proteins, transcriptional regulation

## Abstract

Water temperature stands as a crucial environmental element, exerting an impact on the survival and growth of organisms in aquaculture. Heat stress poses a significant threat to the survival and aquaculture of the Hong Kong oyster *Magallana hongkongensis* (also known as *Crassostrea hongkongensis*), yet the underlying physiological and molecular mechanisms remain poorly understood. This study investigated the effects of elevated temperatures (35 °C and 37 °C) on survival, DNA damage, antioxidant enzyme activities, and gene expression related to apoptosis, inflammation, and heat shock proteins (HSPs) in *M. hongkongensis*. The median lethal temperature (LT50) of *M. hongkongensis* was determined to be 37.09 °C, with significant mortality observed at 35 °C compared with the control (29 °C). Antioxidant enzyme activities (SOD, CAT, and GPx) and T-AOC were up-regulated initially but exhibited divergent patterns under prolonged stress, indicating a temperature-dependent threshold for oxidative defense. Comet assay results also showed that heat stress induced severe DNA damage in hemocytes. Moreover, heat stress significantly up-regulated mRNA expression of apoptosis-related genes (*Caspase-2*, *Caspase-8*, *Bax*, and *P53*), inflammatory genes (*TNF*, *p38-MAPK*, and *AP-1*), and HSP family members (*Hsp70*, *Hsp90*, *Hsp27*, and *Hsp68*). The expression peaks of these genes were generally earlier and more pronounced at 37 °C, reflecting intensified cellular damage and protective responses. Collectively, this study demonstrates that *M. hongkongensis* employs integrated antioxidant, apoptotic, inflammatory, and HSP-mediated mechanisms to counteract heat stress, but temperatures exceeding 35 °C disrupt these defenses, leading to survival impairment. These findings provide critical insights into the heat adaptation strategies of *M. hongkongensis* and serve as a scientific foundation for developing sustainable aquaculture practices to mitigate summer heat stress.

## 1. Introduction

Water temperature is one of the most critical environmental factors affecting aquatic animals. As ectothermic animals, bivalves have their body temperature directly or indirectly determined by water temperature. Water temperature fluctuations significantly influence the physiological and biochemical responses in bivalves, including multiple aspects such as perturbation of metabolic functions [1], induction of tissue structural modifications [2], modulation of lipid and fatty acid profiles [3], and changes in behavioral responses [4]. Once the temperature surpasses the tolerance threshold, the survival rate will be threatened. Summer mortality events associated with high temperatures have occurred in mussels [5,6], oysters [7,8], and scallops [9].

As reported in previous studies, excessive reactive oxygen species (ROS) can be generated when aquatic animals are exposed to heat stress [10,11,12]. This leads to oxidative damage and thus causes DNA damage [13], inflammatory responses [14], and cell apoptosis [15]. Antioxidant enzymes such as superoxide dismutase (SOD), catalase (CAT), and glutathione peroxidase (GPx) can scavenge excessive reactive oxygen species (ROS), maintain the relative balance of oxidation–reduction, and prevent impairment of cellular functions [16,17]. Heat shock proteins (HSPs), which function as molecular chaperones, play a crucial role in maintaining protein homeostasis and collaborate with the antioxidant system to prevent damage caused by heat stress [18,19,20]. The HSPs of various marine animals are activated when they encounter heat conditions [21,22,23]. Discovering the physiological modulation processes is crucial for understanding the adaptation mechanisms of shellfish to heat stress.

The Hong Kong oyster *Magallana hongkongensis* (also known as *Crassostrea hongkongensis*), exclusively distributed along the coastal areas of South China and with a long history of cultivation, is one of the three major cultivated oyster varieties and occupies an important position in modern aquaculture [24,25]. Some Hong Kong oysters are fattened in ponds before being marketed. When heatwaves strike, summer pond water temperatures exceed 35 °C, which poses a serious threat to the survival of *M. hongkongensis*. Understanding the response mechanisms of *M. hongkongensis* to heat stress will help formulate science-based summer aquaculture management measures and promote the sustainable development of the industry.

Hemocytes serve as the key effector components in the immune responses of invertebrates [26]. The gill, an organ with respiratory, osmoregulatory, and immune functions that is directly exposed to the aquatic environment [27], is likely more vulnerable to water temperature variations than other tissues [28]. In this study, we first assessed the survival rate of Hong Kong oysters under high-temperature conditions. Subsequently, we examined the effects of elevated temperatures on DNA damage in oyster hemocytes, as well as the activities of antioxidant enzymes and gene expression related to apoptosis, inflammation, and HSPs in the gills under the high-temperature sensitive threshold and median lethal temperature conditions. This will help depict a comprehensive picture of physiological responses under heat stress and provide a scientific basis for formulating practical solutions.

## 2. Materials and Methods

### 2.1. Animals

Oysters (mean shell height 103.4 ± 6.59 mm) were obtained from an oyster farm in Huidong (Huizhou, China). They were maintained in a recirculating system with a sand filter (salinity 18 ppt, temperature 29 ± 1 °C) for 2 weeks, with oxygen supplied by an air pump. Prior to the experiment, they were fed daily with *Spirulina platensis* powder at 5‰ of their body weight, and half of the water was renewed daily.

### 2.2. Temperature Tolerance Testing

After acclimation, the oysters were randomly assigned to five groups: one control group maintained at 29 °C and four thermal stress groups at 32 °C, 35 °C, 38 °C, and 41 °C (since sessile marine animals inhabiting estuarine intertidal zones may be exposed to high temperatures exceeding 40 °C during low tide [29]), respectively. Each group consisted of 60 individuals and was divided into three replicates. The temperature was increased from 29 °C to the set temperature at a constant rate of 1 °C per hour. The experiment lasted for one week, during which the mortality rate of each group was recorded to determine the high-temperature sensitivity threshold (defined as the lowest temperature that shows a significant difference from the control group) and the median lethal temperature for subsequent heat stress experiments.

### 2.3. Thermal Stress and Sampling

Oysters were heat-stressed at 35 °C (high-temperature sensitivity threshold, T35) and 37 °C (median lethal temperature, T37), with 29 °C as the control (CG). The experiment included three replicates, with a heating rate of 1 °C per hour. At 3 h, 6 h, 12 h, 24 h, 48 h, 72 h, and 96 h after the set temperature was reached, 6 oysters per group (2 oysters per replicate) were sacrificed, and gill tissues were sampled for enzyme activity assays and gene expression analysis. Additionally, on days 1, 2, 3, and 4 after reaching the set temperature, another 6 oysters per group (2 oysters per replicate) were sacrificed, and blood cells were harvested from the heart via a sterile syringe for the detection of DNA damage.

### 2.4. Comet Assay

The comet assay was conducted according to the method provided by Singh et al. [30], with slight modifications. Briefly, blood cells collected on days 1, 2, 3, and 4 after high-temperature stress were diluted with Hanks’ balanced salt solution to a density of 1 × 10^5^ cells/mL. Twenty microliters (20 μL) of the cell suspension were mixed with 50 μL of 0.75% low-melting-point agarose at 37 °C within 2 min and dropped onto frosted glass microscope slides precoated with a 0.5% normal-melting-point agarose layer. The agarose was immediately allowed to solidify at 4 °C for 10 min. The slides were immersed in fresh ice-cold cell lysis buffer (2.5 M NaCl, 100 mM EDTA, 10 mM Tris, 1% Triton X-100, pH 10, 10% DMSO) in the dark at 4 °C for 2 h, then placed in electrophoresis buffer (0.3 M NaOH, 1 mM EDTA, pH > 13) at 4 °C for 20 min to allow DNA unwinding. Subsequently, electrophoresis was performed at 200 mA and 20 V for 20 min. Afterward, slides were neutralized in neutralization buffer (0.4 M Tris-HCl, pH 7.5) for 10 min and dehydrated in ethanol for 15 min. Finally, the slides were stained with SYBR Green and photographed under a fluorescence microscope (Leica, Wetzlar, Germany). The CASP image analysis system [31] was used to measure the Olive Tail Moment (OTM), after counting at least 100 cells per slide.

### 2.5. RNA Extraction, cDNA Synthesis, and Real-Time Quantitative PCR

Total RNA was extracted from gill tissues using Trizol reagent (Invitrogen, Carlsbad, CA, USA) according to the manufacturer’s instructions. The quality of total RNA was evaluated via electrophoresis on 1% agarose gels. Subsequently, 1 μg of total RNA was used to synthesize single-stranded cDNA using the PrimeScript™ RT reagent Kit with gDNA Eraser (Takara, Dalian, China) according to the manufacturer’s protocol. The genes *Hsp70*, *Hsp90*, *Hsp68*, *Hsp27*, *Caspase-2*, *Caspase-8*, *Bax*, *P53*, *TNF*, *p38-MAPK*, and *AP-1* were analyzed, and the GAPDH gene was chosen as the reference gene. All primer sequences are listed in Table 1. qRT-PCR was performed on a Qtower96G real-time system (Analytik Jena AG, Jena, Germany) using the SYBR Green Premix Pro Taq HS qPCR Kit (Accurate Biology, Guangzhou, China) according to the manufacturer’s instructions. The amplification protocol was as follows: 95 °C for 5 min, 40 cycles at 95 °C for 5 s and 60 °C for 30 s, followed by dissociation curve analysis. Three biological replicates were included in the experiments, and each sample was examined three times on the same plate. The relative expression levels of the target genes were calculated as fold changes by normalizing to *β-actin* and *GAPDH*, using the 2^−ΔΔCt^ method [32].

### 2.6. Determination of Enzyme Activities Related to the Antioxidant System

The gill tissues collected at each time point were homogenized in 10 volumes (*v*/*w*) of pre-cooled sterile 0.9% saline solution. The homogenates were centrifuged at 2500 rpm for 10 min at 4 °C. The resulting supernatants were then used for antioxidant enzyme analysis. The protein content of each gill sample was determined using the Coomassie Brilliant Blue method. The activities of catalase (CAT), superoxide dismutase (SOD), glutathione peroxidase (GPx), and total antioxidant capacity (T-AOC) were measured according to the protocols of commercial assay kits (Nanjing Jiancheng Sci-Tech Co., Ltd., Nanjing, China).

### 2.7. Statistical Analysis

All data were expressed as means ± standard deviation (SD). In temperature tolerance tests, statistical differences in survival rates under different heat stress temperatures were determined using one-way ANOVA followed by Duncan’s multiple range tests. Similarly, in other experiments, significant differences between groups at the same time points were analyzed using the same statistical approach (one-way ANOVA followed by Duncan’s multiple range tests). Statistical analysis was performed with SPSS 18.0 software (SPSS Inc., Chicago, IL, USA). Differences were considered significant at *p* < 0.05.

## 3. Results

### 3.1. The Effects of High Temperature Stress on the Survival Rate of Hong Kong Oysters

**Figure 1:**
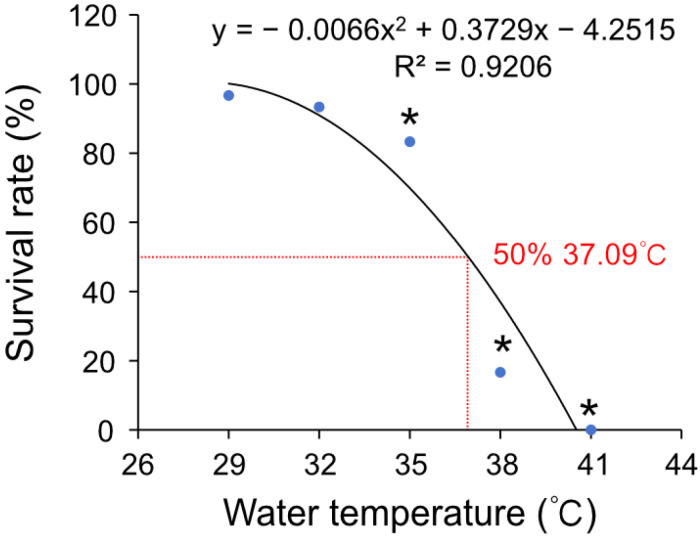
The fitting curve of survival rate at high temperature of Hong Kong oysters. The significant differences in survival rates under different heat shock temperatures were determined by one-way ANOVA followed by Duncan’s multiple range tests (* *p* < 0.05).

To investigate the impact of high temperature on the survival rate of Hong Kong oysters, a series of equally spaced temperatures was selected. With the increase in temperature stress, the survival rate of the oysters declined precipitously (Figure 1). Multiple comparisons revealed that significant differences from the control group began at 35 °C (*p* < 0.05). Using regression analysis, the median lethal temperature for Hong Kong oysters was calculated as 37.09 °C. Following this analysis, 35 °C and 37 °C were selected as high-temperature stress temperatures for subsequent experiments.

### 3.2. DNA Damage After Heat Stress

**Figure 2:**
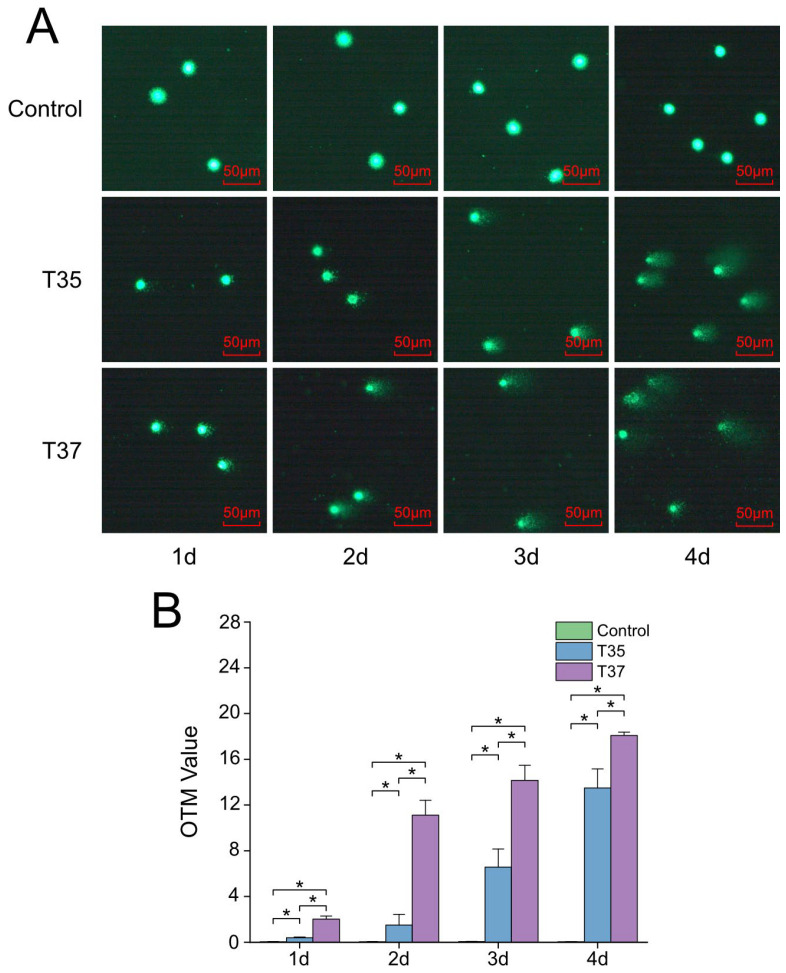
DNA damage in hemocytes of Hong Kong oysters under heat stress assessed by comet assay. (**A**) Representative comet images of hemocytes from oysters exposed to 29 °C (control), 35 °C, and 37 °C. (**B**) Olive tail moment (OTM) values under each temperature condition. Asterisks indicate significant differences (*p* < 0.05).

A comet assay was performed to assess the effect of heat stress on the DNA damage of Hong Kong oysters’ hemocytes. As shown in Figure 2A, the tail length became increasingly pronounced as time progressed under heat stress. The 37 °C group exhibited a significantly higher OTM value than both the 35 °C group and the control group throughout the experiment (Figure 2B).

### 3.3. Changes in Antioxidant Enzyme Activity in Response to Heat Stress

**Figure 3:**
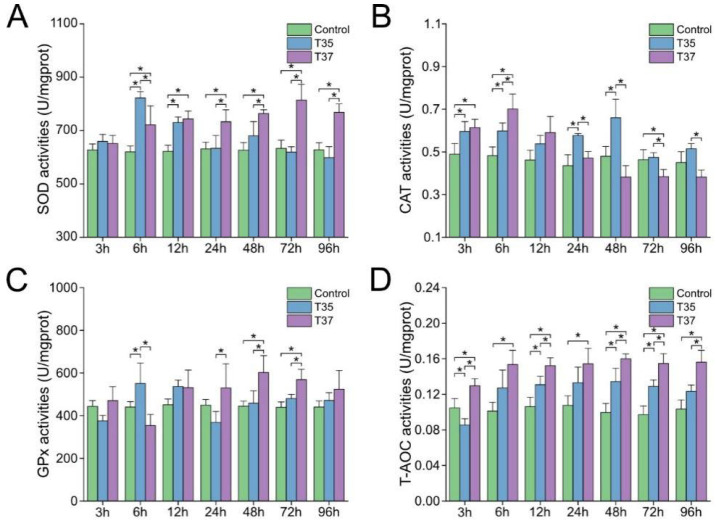
Effects of heat stress on antioxidant enzyme activity in gills. (**A**) SOD activity. (**B**) CAT activity. (**C**) GPx activity. (**D**) T-AOC activity. Data are presented as the mean ± SD. Asterisks indicate significant differences between groups at the same time points, as determined by Duncan’s multiple range test.

The SOD activity increased in the heat stress groups and was significantly higher than that of the control group at 6 h and 12 h (*p* < 0.05). Afterward, the SOD activity of the T35 group decreased, while the T37 group maintained significantly higher activity (Figure 3A). For CAT activity, the activities of the T35 and T37 groups were significantly higher than those of the control group at 3 h and 6 h after heat stress (*p* < 0.05). Afterward, the T35 group showed significantly higher activity than both the control group and the T37 group at 24 h and 48 h (*p* < 0.05), while the T37 group recovered to normal levels (Figure 3B). In the case of GPx activity, the T35 group was significantly higher than the control and T37 groups at 6 h (*p* < 0.05), while the T37 group demonstrated significantly higher activity than the other two groups at both 48 h and 72 h (*p* < 0.05) (Figure 3C). The T-AOC activity is shown in Figure 3D. The activity of both the T35 and T37 groups increased at 6 h and remained stable. Although the activity of the T35 group was higher than that of the control group after 6 h, significant differences from that of the control group were only observed at 12 h, 48 h, and 72 h. In contrast, the T37 group was significantly higher than the control group at all time points.

### 3.4. Changes in mRNA Expression of Apoptosis-Related Genes

**Figure 4:**
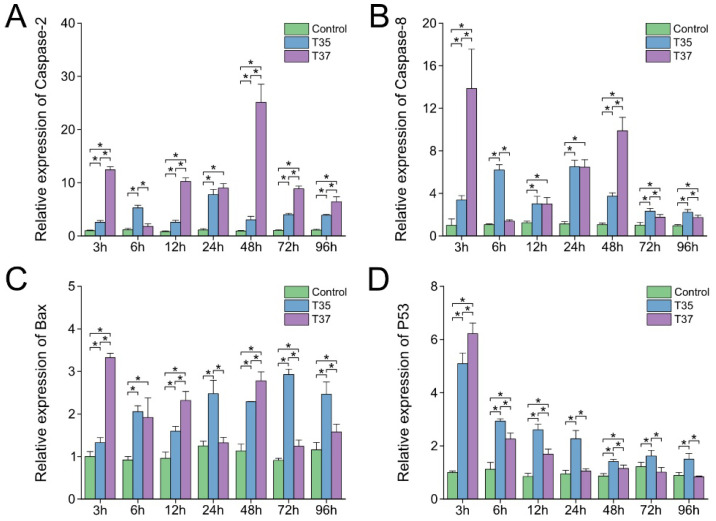
Effects of heat stress on apoptosis-related genes in gills. (**A**) *Caspase-2*. (**B**) *Caspase-8*. (**C**) *Bax*. (**D**) *P53*. Data are presented as the mean ± SD. Asterisks indicate significant differences between groups at the same time points as determined by Duncan’s multiple range test.

The relative expression levels of apoptosis-related genes *Caspase-2*, *Caspase-8*, *P53*, and *Bax* after heat stress were detected by qPCR (Figure 4). As shown in Figure 4A–C, although the expression levels of *Caspase-2*, *Caspase-8*, and *Bax* showed fluctuating patterns, they were significantly higher than those in the control group at multiple time points (*p* < 0.05). Regarding *P53*, both the T35 and T37 groups exhibited peak expression at 3 h after heat stress. Subsequently, a decrease in expression was observed in the T37 group, which remained stable after 24 h and showed no significant difference from the control group. Although the T35 group also showed a downward trend and remained stable starting from 48 h, its expression level was higher than that of both the control group and the T37 group at all time points from 6 h onward (Figure 4D).

### 3.5. Changes in mRNA Expression of Inflammation-Related Genes

**Figure 5:**
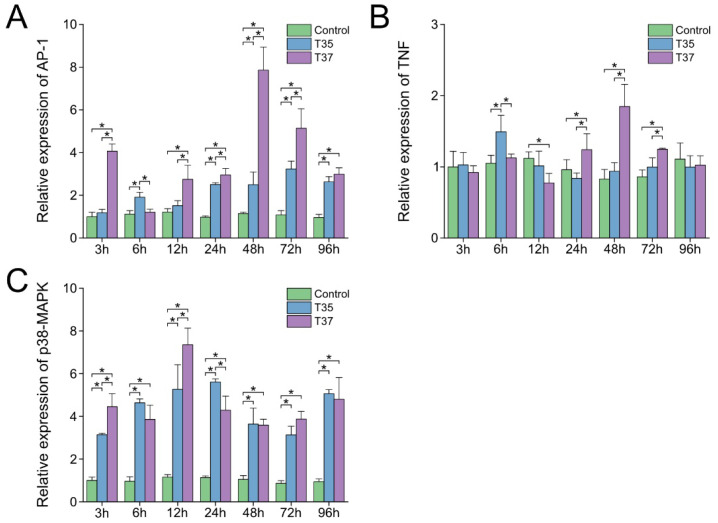
Effects of heat stress on inflammation-related genes in gills. (**A**) *AP-1*. (**B**) *TNF*. (**C**) *p38-MAPK*. Data are presented as the mean ± SD. Asterisks indicate significant differences between groups at the same time points as determined by Duncan’s multiple range test.

The changes in inflammation-related gene expression in gills under heat stress are presented in Figure 5. Beginning at 24 h post-heat stress, *AP-1* expression in both treatment groups was markedly elevated compared with that in the control group (*p* < 0.05), while *p38-MAPK* expression in the treatment groups was significantly higher than in the control group at all time points following heat stress (*p* < 0.05). In the T35 group, the expression levels of *AP-1* and *p38-MAPK* reached their peaks at 72 h and 24 h, respectively. In the T37 group, they reached their peaks at 48 h and 12 h, with peak levels higher than those in the T35 group (Figure 5A,C). The mRNA expression of *TNF* in the T35 group was significantly higher than that in other groups at 6 h (*p* < 0.05). In contrast, the T37 group showed significantly higher expression than other groups at 24–72 h (*p* < 0.05) (Figure 5B).

### 3.6. Changes in mRNA Expression of HSP Member Family Genes

**Figure 6:**
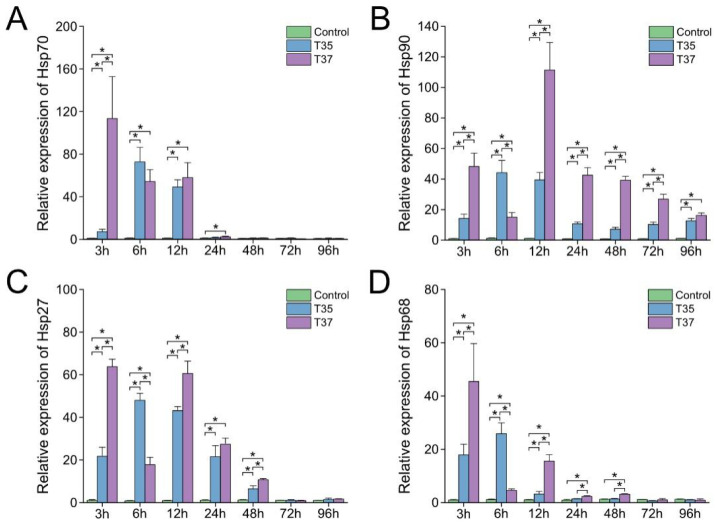
Effects of heat stress on Heat Shock Protein (HSP) family genes in gills. (**A**) *Hsp70*. (**B**) *Hsp90*. (**C**) *Hsp27*. (**D**) *Hsp68*. Data are presented as the mean ± SD. Asterisks indicate significant differences between groups at the same time points as determined by Duncan’s multiple range test.

As illustrated in Figure 6, under heat stress, both experimental groups exhibited up-regulation of *Hsp70* and *Hsp68* transcripts from 3 h to 12 h, *Hsp90* transcripts from 3 h to 96 h, and *Hsp27* transcripts from 3 h to 48 h (*p* < 0.05). In the T35 group, all detected heat shock protein genes reached their maximum expression levels at 6 h. In contrast, in the T37 group, the maximum expression of *Hsp70*, *Hsp27*, and *Hsp68* was observed at 3 h, whereas the peak expression of *Hsp90* occurred at 12 h post-treatment. Notably, among the time points when both experimental groups exhibited significantly higher expression levels than the control group, the heat shock protein gene expression levels in the T37 group were higher than those in the T35 group at all time points except 6 h.

**Figure 7:**
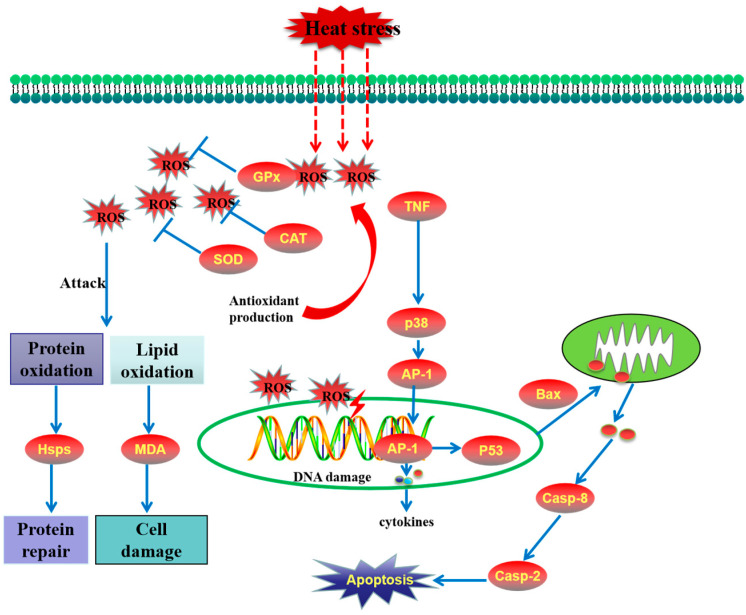
The schematic model of heat stress-induced toxicity in *Magallana hongkongensis*.

We also demonstrated the schematic model of heat stress-induced toxicity in *M. hongkongensis* (Figure 7).

## 4. Discussion

As a vital abiotic element, water temperature influences the physiological state of aquaculture animals [36,37]. Bivalves have evolved the capacity to endure temperature changes within a specific range [38,39], yet extreme heat can still threaten their survival [40]. *M. hongkongensis* is a key aquaculture shellfish along the coast of South China, particularly in Guangdong and Guangxi, where it accounts for 60–71% of total shellfish production (including a small proportion of *Crassostrea angulata*). Studying its survival rate and physiological responses to high temperature is crucial for formulating preventive management measures. In this study, the survival rate of *M. hongkongensis* started to differ significantly from the control group at 35 °C and dropped to 50% at around 37.09 °C. Some studies indicate that oysters can tolerate drastic daily and seasonal temperature fluctuations [41] and exhibit a higher thermal stress tolerance than subtidal species. A comparative study on the responses of immunological parameters to temperature changes in three species of marine bivalves revealed that the activities of SOD and ROS in the hemocytes of Mediterranean mussels and mud cockles increased with rising temperatures, whereas the increases in ROS and SOD in Pacific oysters only occurred within the range of 20 °C to 25 °C [42]. Rajagopal et al. [43] studied the time required for *Crassostrea gigas* to reach 100% mortality at different temperatures and compared this with that of other shellfish. They pointed out that the upper limit of heat tolerance of *C. gigas* is much higher than that of other major marine fouling animals, including *Mytilus edulis*. Some studies have indicated that the enhanced heat tolerance stems from the induction of heat shock proteins [44]. Analysis of the oyster genome shows that the heat shock protein family of *C. gigas* has undergone expansion, with a notably larger number of coding genes than other animals, a feature that partially explains the tolerance of *C. gigas* to high temperatures [45]. However, oysters are ectothermic animals, meaning their body temperature quickly synchronizes with the water temperature during aquaculture. Unable to escape adverse effects, they may eventually die under extremely high temperatures. Thus, effective artificial preventive measures are essential.

Hemocytes of mollusks perform crucial physiological functions. They participate not only in innate immune defense [46] but also in functions like nutrient transportation [47], tissue repair [48], and shell mineralization [49]. DNA stores genetic information and dominates key life processes. However, its bases can be modified by excessive reactive oxygen species (ROS) generated by environmental stress, leading to DNA strand breakage via ribose ring degradation. In this study, the comet assay, a rapid method for detecting DNA damage, was used to assess DNA damage in hemocytes of Hong Kong oysters under heat stress. The results demonstrate that high temperature induces DNA damage in hemocytes, which exhibits dependence on both temperature and time. Previous studies have shown that high temperatures can cause DNA damage in other aquatic animals, such as freshwater crayfish [50], fish [13], and sea cucumber [51]. This implies that heat stress can exert genotoxic effects on aquatic species.

Heat stress can induce the production of reactive oxygen species (ROS), leading to oxidative stress and the disruption of redox homeostasis [52,53]. Antioxidant enzymes, such as SOD, CAT, and GPx, serve as the initial defenses against ROS. These antioxidant enzymes each perform specific functions and ultimately convert superoxide radicals into non-toxic water to promote cellular homeostasis maintenance and alleviate oxidative stress [22,54]. In this study, the activities of SOD, CAT, and GPx increased significantly after heat shock, which is consistent with previous studies on *Perna viridis* [40] and *Mytilus galloprovincialis* [12]. In addition, total antioxidant capacity (T-AOC), which consists of enzymatic and non-enzymatic antioxidants and reflects the overall antioxidant ability of organisms [55], also increased significantly under heat stress. Notably, the T37 group showed a higher T-AOC level than the T35 group, suggesting that 37 °C induces more severe oxidative damage in *M. hongkongensis*.

Heat stress causes oxidative damage and induces cellular apoptosis by regulating the expression of apoptosis-related genes. P53, Bax, Caspase-2, and Caspase-8, as key apoptosis-related molecules, are involved in the apoptotic response. As a key regulatory factor, P53 exerts antioxidant functions to safeguard cells under low oxidative stress while promoting oxidation [56,57,58] and triggering apoptosis to remove damaged cells under high oxidative stress [59]. DNA damage can activate P53, which induces the expression of Bax and thereby triggers the release of cytochrome c from mitochondria [60]. The released cytochrome c activates caspases, which in turn drive cells toward apoptosis [61]. Caspase-2 and Caspase-8 function as initiator caspases, with Caspase-2 being crucial for DNA damage-induced apoptosis [62,63] and Caspase-8 activation triggering downstream effectors (Caspase-3, -6, and -7), which lead to the hallmark features of apoptosis [64]. It has been reported that acute heat stress induces hemocyte apoptosis in the Pacific oyster [65]. Additionally, the transcription of *p53* and *Bax* was up-regulated in pufferfish and turbot following heat stress [22,66]. In this study, *p53*, *Bax*, *Caspase-2*, and *Caspase-8* were markedly induced, suggesting that the p53–Bax pathway and the caspase-dependent apoptotic pathway were involved in heat stress-induced apoptosis in Hong Kong oysters.

Inflammatory manifestations represent the secondary response elicited by oxidative stress [67]. TNF-α is a typical pro-inflammatory cytokine that initiates and mediates inflammatory processes [68,69]. TNF-α is a potent activator of p38-MAPK [70], which in turn regulates the transcription of *AP-1* [71]. During inflammation, cytokines and chemokines orchestrate immune cell attraction and activation, whereas AP-1, a pivotal transcription factor, modulates the expression of these cytokines and chemokines [72,73]. It has been reported that thermal stress induces transcriptional up-regulation of *TNF-α* and elicits an inflammatory response in gilthead seabream and pikeperch [74,75]. Concomitantly, heat stress promotes phosphorylation of p38-MAPK in the green mussel and augments *AP-1* transcription in corals and sea urchins [12,76,77]. In this study, the up-regulated expression of *TNF*, *p38-MAPK*, and *AP-1* in Hong Kong oysters indicates that heat stress activated the inflammatory signal transduction pathway.

Heat shock proteins, which act as pivotal regulators in the heat stress response, can associate with antioxidant enzymes to scavenge generated ROS [78] and protect against oxidative stress [79]. The up-regulation of HSPs appears to be a phylogenetically conserved mechanism utilized by aquatic organisms to counteract thermal stress, which can be found in fish [80], crustaceans [78], and shellfish [81]. Expression levels of HSPs are considered molecular bioindicators for monitoring thermal stress [82,83]. In this study, enhanced transcription of *Hsp70*, *Hsp90*, *Hsp68*, and *Hsp27* was observed after heat stress, consistent with previous reports. Compared with the T35 group, the T37 group exhibited higher expression levels of HSPs. This finding not only suggests substantial protein damage in the T37 group but also indicates a more pronounced activation of protective mechanisms.

In this study, it can be observed that both high-temperature stress conditions induce physiological responses in oysters, and the stress response at 37 °C is generally stronger than that at 35 °C. However, it is noteworthy that after 6 h of high-temperature stress, the immune response of the T35 group is higher than that of the T37 group in terms of multiple indicators. A possible reason for this is that the damage to oysters under 37 °C is more severe, leading to the production of a large number of misfolded proteins. In the T37 group, multiple indicators increased significantly after 3 h of stress exposure, especially HSPs. HSPs require energy supplied by ATP to help these proteins regain their natural conformation and biological activity [84]. Additionally, the synthesis of HSPs also consumes energy, but excessive energy consumption may compromise the physiological and immune statuses of oysters [29]. Under the regulation of a balancing mechanism, the immune intensity of the T37 group decreased at 6 h. In contrast, the stress condition of 35 °C is milder, and the immune intensity increased gradually, resulting in the immune intensity of the T35 group being higher than that of the T37 group at 6 h.

## 5. Conclusions

In conclusion, *M. hongkongensis* employs a complex network of antioxidant defense, apoptotic, inflammatory, and HSP-mediated responses to cope with heat stress (Figure 7). However, the species exhibits limited tolerance to temperatures above 35 °C, emphasizing the need for proactive management strategies (e.g., temperature control and nutritional supplementation) to prevent summer mortality. These findings not only advance our understanding of bivalve heat adaptation but also offer practical implications for sustainable aquaculture of *M. hongkongensis*.

## Figures and Tables

**Table 1:** The sequences of primers in this study.

Primer Name	Primer Sequence (5′-3′)	Expected Size (bp)	Efficiency (%)	Gene Accession Number or Source
Hsp70-F	CACCACATACTCCGATAACCA	255	97.3	FJ157365.1
Hsp70-R	GCCTTACTCAGACGACCTTTA			
Hsp90-F	TTGAAAAGGTGGTGGTATCTAAC	298	98.1	HM171376.1
Hsp90-R	CCTGGCTCCTCCAAACTAAA			
Hsp68-F	GGACGCAGGTTTACTGACGA	229	96.2	GU586491.1
Hsp68-R	TGAAATAGGCTGGCACGGT			
Hsp27-F	ACAAAGACCTATCTGCTTCCTG	128	99.5	Li et al. [33]
Hsp27-R	ATCACTCTCTCCTTCGGGG			
Caspase-2-F	AGATAAAATCCACAGGAGCAAT	164	97.6	ON803398.1
Caspase-2-R	CAGAAACCAGGACCCGTAT			
Caspase-8-F	GATGGACCAAACTATCTTACCG	217	98.5	KC822928.1
Caspase-8-R	GCTGTTCGAGTGTCTTCACG			
Bax-F	CACACCCACAGGTCCTCCAC	185	99.1	KM262836.1
Bax-R	CCCAGTTGTAAACACCATCAGC			
P53-F	GAGTCAACGCCAACCACC	152	97.8	MZ420636.1
P53-R	TCACCAAATGAGTCGGAGC			
TNF-F	GAGACATCGCCTTCATCAGC	130	98.7	KX698405.1
TNF-R	TGCGTGCCTCCACTACTTC			
p38-MAPK-F	GGAACCCCTAACCAGGCACTT	220	97.5	KX698406.1
p38-MAPK-R	CAGCATACTGAGCCAAATACGG			
AP-1-F	CGGGTTCGTGGAGGCAT	158	99.2	KC890768.1
AP-1-R	CGTCAGTGTTGGAATAGGAGGA			
GAPDH-F	GGATTGGCGTGGTGGTAGAG	184	99.3	He et al. [34]
GAPDH-R	GTATGATGCCCCTTTGTTGAGTC			
β-actin-F	CTGTGCTACGTTGCCCTGGACTT	129	99.5	She et al. [35]
β-actin-R	TGGGCACCTGAATCGCTCGTT			

## Data Availability

Data are contained within the article.
